# Vertebrae Destruction with Cauda Equina Syndrome Secondary to Spinal Gouty Arthritis: A Case Report

**DOI:** 10.5704/MOJ.2007.024

**Published:** 2020-07

**Authors:** K Thuraikumar, KL Wan, KL Ong, SW Lim

**Affiliations:** Department of Orthopaedics and Traumatology, Hospital Sungai Buloh, Sungai Buloh, Malaysia

**Keywords:** spinal gouty arthritis, spinal gouty tophi, spine gout, spinal canal stenosis secondary to gout, vertebral destruction secondary to gout

## Abstract

Gouty arthritis commonly affects peripheral joints and is associated with hyperuricaemia. Spinal manifestations of gouty arthritis are not common, and majority of published articles worldwide were case reports. This is a case report of spinal gouty arthritis that presented with spinal vertebrae destruction and cauda equina syndrome. The magnetic resonance imaging (MRI) showed destruction of L5/S1end plates with cystic collection mimicking infective changes. The tissue histological examination confirmed presence of urate crystal needles that displayed negative double refraction on light microscopy. Spinal gouty arthritis is part of the differential diagnoses in gouty arthritis patients.

## Introduction

Gout is a metabolic disease associated with hyperuricaemia and typically presents with inflamed peripheral joints. Nucleic acids and purines are metabolised to eventual uric acid which is excreted 90% via the kidney and 10% via the intestine^1, 2^. The uric acid forms monosodium urate crystal that can invade into connective tissues and potentially increase in size to form tophus. This urate crystal causes acute inflammation of the affected joint and in chronicity, leads to progressive destruction of the joint.

We present a rare case of spinal gouty arthritis that caused vertebrae destruction and cauda equina syndrome.

## Case Report

**Fig. 1:**
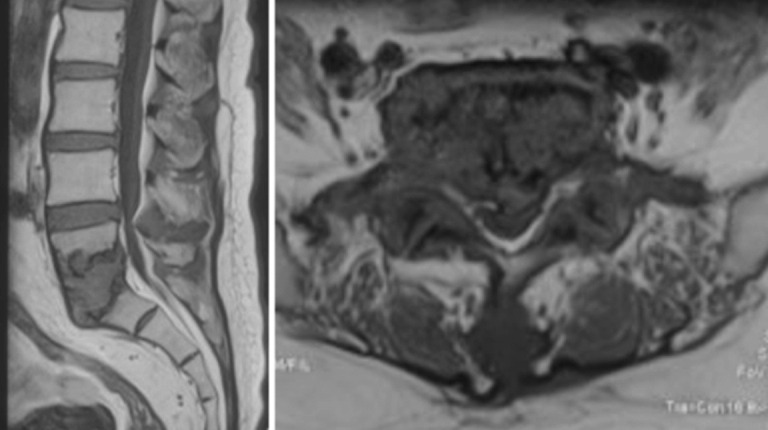
Magnetic resonance imaging (MRI) T1 weighted of sagittal and axial views which showed irregular inferior end plate L5 and superior end plate S1, expanded.

**Fig. 2:**
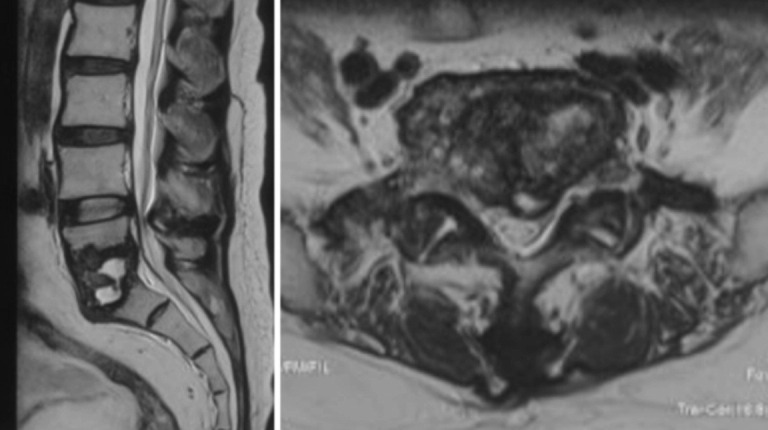
Magnetic resonance imaging (MRI) T2 weighted sagittal view showed cystic lesions without adjacent marrow oedema under the irregular end plates of L5 and S1.

The patient was a 68-year-old gentleman who had underlying co-morbidities of gouty arthritis on allopurinol, hypertension and diabetes mellitus. His medication compliance was not optimum, and he had been experiencing episodes of gout exacerbation. He presented with chronic low back pain for a year. He developed progressive worsening of lower limb weakness six months prior to his presentation. His cauda equina symptoms with intermittent loss of urinary and bowel controls developed gradually over recent six weeks. At presentation, his lower limbs power was grade two and he had saddle anaesthesia with laxed anal tone. Magnetic resonance imaging (MRI) showed end plates destruction of L5/S1, partial vertebral bodies destruction, cystic fluid collection, anterior longitudinal ligament (ALL) subligamentous spread and central spinal canal stenosis as shown in Fig. 1 (T1 weighted image) and Fig. 2 (T2 weighted image). C-reactive protein (CRP) was 2.37 mg/dL (slightly elevated), erythrocyte sedimentation rate (ESR) 64 mm/hour, total white blood cell (WBC) 10.2 x109/L, uric acid 371 μmol/L (normal) and alkaline phosphatase (ALP) 167 U/L (slightly elevated but other liver enzymes were within normal ranges). Infective spondylodisciitis of L5/S1 was suspected.

**Fig. 3:**
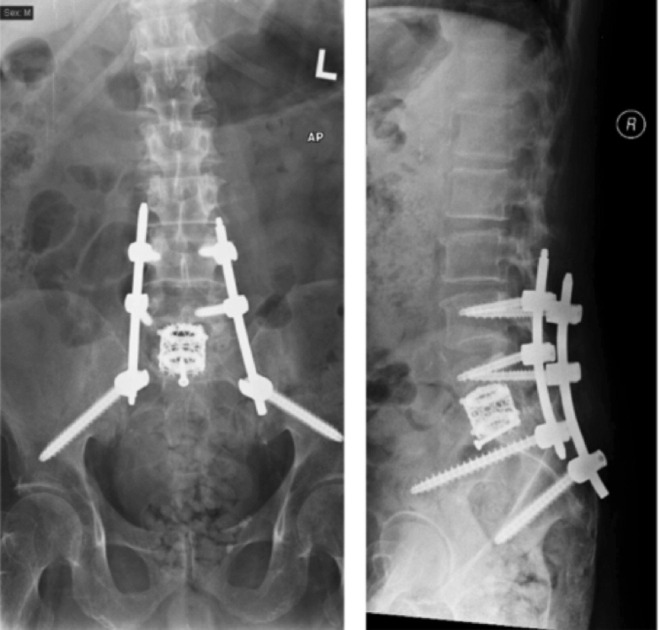
Post-operative radiographs of anteriorposterior and lateral views of lumbo-sacral spine.

A two-stage operation was done. The first stage was the anterior approach for L5/S1 vertebrae partial corpectomy, and lumbar interbody titanium mesh cage was inserted. The second stage involved a posterior approach for lumbo-pelvic instrumentation and fusion. Iliac screws were inserted instead of S1 in view of the osteoporotic quality of S1 vertebrae. Demineralised bone matrix (DBM) bone allograft was used for fusion purposes. A whitish lump of tissue from the vertebrae was sent for histological examination, of which the report was consistent with gouty tophi with adjacent thick fibrocollagenous connective tissue. The crystals within the tophus were confirmed with polarised light microscope and displayed negative double refraction. No granuloma nor malignancy was found in the histology. Tuberculosis and fungal cultures did not grow any organisms. The diagnosis was then changed to spinal gouty arthritis.

His urinary and bowel controls returned to full function after three months. His lower limbs power improved to grade four after six months. He did not report of any instability spinal symptoms and lumbo-sacral radiographs did not show subsidence. However, a longer follow-up with repeated MRI and CT might be necessary to assess recurrence and fusion quality.

## Discussion

Spinal gouty arthritis is rare and is mostly reported only as case reports. Konatapalli *et al* reported 35% incidence of spinal gouty arthritis on computed tomography (CT) scan in a three-year prospective study but only about half had spinal symptoms^1^. The author also found that most cases involved lumbar spine (56%) whilst cervical and thoracic spine contributed to 22% of the incidence1. The facet joints would be assumed to be affected earliest and to have the worst destruction. This is due to their ‘resemblance’ of peripheral joints that are common sites for gouty arthritis presentation. However, the exact pathophysiology of spinal gouty arthritis is unclear. Urate crystal formation is favored in acidic environment and lower temperature - both factors reduce protein binding affinity to urate and increase the rate of urate crystal deposition. This explains the higher preponderance towards peripheral joints tophi formation^2^. However, lower temperature is not relevant to spinal joints as the temperature is closer to the core body temperature. It is postulated that any event that induces inflammation process such as trauma and degenerative condition may initiate an acidic environment which leads to urate crystal deposition and lower solubility of the crystal^2^. As a result, the location of tophi formation could be anywhere in the spine such as vertebra bodies, facet joints, epidural space and intervertebral disc^3^. The clinical presentation of spinal gouty arthritis is, therefore, variable anatomically and its progression depends on the dynamic gouty arthritis exacerbations. Wendling *et al* reported that 39% of patients presented with weakness, 27% with radiculopathy and 18% with backpain^3^.

Imagings are not specific for spinal gouty arthritis^4^. The changes in plain radiographs, CT and MRI depend on the severity of crystal deposition, inflammation and surrounding tissue destruction. The dynamic natural history of gouty arthritis also makes all imaging changes to be non-specific. Plain radiographs and CT may show vertebral bodies destruction, end plates erosions and listhesis, which could all point to tumour or infective changes. A gouty tophus usually shows a mass with hypointense signal in T1 and T2 weighted sequences and may have increased gadolinium uptake during its exacerbation due to hyperinflammatory changes to the surrounding tissue^4^. Post inflammation processes may involve cystic formation, calcification and new tophus formation which mimic tumourous condition^4^. A tissue biopsy is the gold standard in diagnosing a spinal gouty arthritis where the polarised microscopy confirms the negatively birefringent crystals.

A biopsy is recommended prior to surgical treatments especially in imagings that resemble a tumourous condition^5^. However, appropriate surgical treatments such as urgent decompression and stabilisation for acute neurological deficit supercedes the biopsy confirmation. Medical therapy must be optimised to reduce the recurrence and progression of spinal gouty arthritis. Clinical Practice Guidelines (CPG) for management of gout recommended for serum uric acid to be below 360 μmol/L in erosive gouty arthritis. In certain early conditions, surgical intervention may be avoided with quiescence of gouty arthritis progression.

In conclusion, spinal gouty arthritis is included in the differential diagnoses for patients who have background history of gouty arthritis. The diagnostic confirmation remains challenging without a biopsy. Further studies with longer follow-ups will be important to assess the recurrence and spinal fusion rates in operated spinal gouty arthritis cases.
